# Heart Function Changes During Environmentally Relevant Combined Exposure to Heat, Ultrafine Carbon Black, and Ozone: Role of NOD-like Receptor X1

**DOI:** 10.3390/cells15181699

**Published:** 2026-09-19

**Authors:** Chiara Monge, Yulu Li, William Travis Goldsmith, Atefeh Razazan, Murugesan Velayutham, Ethan Meadows, Mark Eminhizer, John M. Hollander, Salik Hussain

**Affiliations:** 1 Department of Physiology, Pharmacology & Toxicology, School of Medicine, West Virginia University, Morgantown, WV 26506, USA; chiaramonge06@gmail.com (C.M.); yl00041@mix.wvu.edu (Y.L.); wgoldsmi@hsc.wvu.edu (W.T.G.); atefeh.razazan@hsc.wvu.edu (A.R.); jhollander@hsc.wvu.edu (J.M.H.); 2 Center for Inhalation Toxicology (iTOX), School of Medicine, West Virginia University, Morgantown, WV 26506, USA; 3 Mitochondria, Metabolism & Bioenergetics Working Group, School of Medicine, West Virginia University, Morgantown, WV 26506, USA; murugesan.velayutham@hsc.wvu.edu (M.V.); emmeadows22@gmail.com (E.M.); mark.eminhizer@hsc.wvu.edu (M.E.); 4 Department of Biochemistry and Molecular Medicine, School of Medicine, West Virginia University, Morgantown, WV 26506, USA; 5 Department of Microbiology, Immunology and Cell Biology, School of Medicine, West Virginia University, Morgantown, WV 26506, USA

**Keywords:** cardiac function, mitochondrial dysfunction, echocardiography, air pollution, inflammation, extreme heat, NLRX1, bioenergetics

## Abstract

**Highlights:**

**What are the main findings?**

- Deletion of NOD-like receptor X1 (NLRX1) impairs the heart’s ability to adapt physiologically after heat exposure. Combined heat and air pollution exposure results in greater lung inflammation compared to individual exposures.
- NLRX1 deficiency exacerbates lung inflammation and worsens cardiac dysfunction and dyssynchrony during simultaneous extreme heat and air pollution exposure. This combined exposure leads to alterations in cardiac mitochondrial coupling and maximum complex activity.

**What are the implications of the main findings?**

- These findings delineate that combined heat stress and air pollution have a greater propensity to drive lungs and heart dysfunction, and thus people living in more polluted areas may be at greater risk for adverse outcomes.
- NLRX1 is a potential therapeutic target to mitigate the increased cardiopulmonary risk associated with combined heat and air pollution exposure.

**Abstract:**

Extreme heat events are increasing in frequency, duration, and severity, often with higher air pollution (AP) levels. The biological mechanisms underlying combined heat and AP exposures and the role of NLRX1 in pulmonary and cardiac dysfunction remain largely undefined. *Nlrx1^+/+^* and *Nlrx1^−/−^* female mice were exposed to filtered air/CTR, HT (36 °C), ultrafine carbon black (UfCB) + ozone (O_3_) (CB 2.5 mg/m^3^ + O_3_ 1 ppm), and HT + UfCB + O_3_ for 3 h. Pulmonary inflammation was evaluated by bronchoalveolar lavage, while cardiac function and strain were evaluated using Doppler echocardiography. Mitochondrial function was assessed through coupling assay and ETC complex activity measurements. Both at 2 and 24 h, *Nlrx1^−/−^* mice showed no adaptive physiological cardiac responses and demonstrated impaired mitochondrial function after heat-alone exposure. UfCB + O_3_-alone exposure induced a decrease in stroke volume and cardiac output and a decrease in cardiac mitochondrial complex III–V activities in both genotypes. Combined HT + UfCB + O_3_ resulted in a greater reduction in ejection fraction percentage and fractional shortening percentage, greater pulmonary neutrophilia, and a reduction in complex III–V activities at 24 h post-exposure in *Nlrx1^−/−^* mice compared to *Nlrx1^+/+^* mice. Moreover, *Nlrx1^−/−^* mice demonstrated a greater decrease in longitudinal and circumferential strain, indicating dyssynchronous contraction throughout the heart layers and mid-cavity segments. Collectively, our findings indicate that NLRX1 deficiency alters the acute physiological cardiopulmonary responses to HT and UfCB + O_3_.

## 1. Introduction

Extreme heat events have emerged as a major public health threat, with their frequency, duration, and severity increasing due to long-term shifts in global weather patterns and rising average temperatures [1]. The year 2024 set a record for the warmest conditions ever observed and was the first year in which the global average surface temperature surpassed the pre-industrial baseline by more than 1.5 °C [2,3]. In both the United States and Europe, heat waves are now recognized as the leading cause of mortality and heat-related hospital admissions among extreme weather events [4,5,6,7,8]. Heat waves increase hospitalization and mortality, mainly from cardiovascular, cerebrovascular, and respiratory diseases, with effects emerging shortly after onset [3,9,10,11]. A global analysis estimated approximately 490,000 heat-related excess deaths annually during heat waves [12]. This is accompanied by a 15% increase in cardiovascular mortality [13]. The adverse impacts of extreme heat events have also been linked to simultaneous increases in air pollutant levels [14,15,16,17]. Higher temperatures often coincide with increased air pollution, as heat and drought elevate emissions from wildfires and waste decomposition, raising levels of particulate matter and ground-level ozone (O_3_) [18]. Observational and experimental studies consistently demonstrate that exposure to air pollution significantly increases the risk of cardiovascular diseases, including coronary artery disease, stroke, and myocardial infarction [19,20,21]. Notably, the hazardous effects of air pollution are closely linked to environmental temperature, with ambient heat and air pollution exposures capable of mutually exacerbating each other’s adverse health impacts [22,23,24]. Epidemiological studies point towards worsened cardiovascular outcomes, including myocardial infarction and mortality from cardiovascular causes, from combined HT and AP exposure [25,26,27,28]. A synergistic association between ambient heat and PM_2.5_ in terms of out-of-hospital cardiac arrests was observed, indicating a need for integrative risk assessment [29]. Nevertheless, the impact of combined heat and pollutant stress on cardiac physiology, along with the cellular mechanisms mediating these effects, remains poorly understood [22,30,31].

Among potential cellular pathways mediating environmental stress responses, NLRX1 (also known as NOD9) stands out for its multifunctional roles across a range of cellular processes, including inflammation, tissue injury, and innate immune responses. NLRX1 has been identified as an important modulator of mitochondrial function, reactive oxygen species (ROS) generation, autophagy, and apoptosis [32,33,34,35,36,37]. Specifically, it negatively regulates multiple immune pathways, including type-I interferon (IFN) signaling, inflammasome activation, double-stranded RNA (dsRNA)–activated kinase PKR, and pro-inflammatory nuclear factor kappa B (NF-κB) signaling [38,39,40]. Emerging evidence also highlights NLRX1 as a protective factor in various cardiac pathologies such as cardiac ischemia–reperfusion injury by inhibiting MAVS-dependent NLRP3 inflammasome activation [41,42]. Additionally, NLRX1 mitigates cardiac hypertrophy by reducing endoplasmic reticulum stress (ERS) [38]. Very little is known about how heat exposure influences NLRX1 regulation. However, the precise role of NLRX1 in regulating cardiac function during combined exposure to air pollution and heat stress, as well as the cellular mechanisms mediating these effects, remains unclear.

This study aims to address this knowledge gap by employing an environmentally relevant co-exposure model using a mixture of ultrafine carbon black (UfCB) and ozone (O_3_) as an air pollution surrogate (UfCB + O_3_), together with high temperature (HT) exposure, to elucidate the role of NLRX1 deficiency in cardiac functional responses. We hypothesize that NLRX1 plays a protective role in combined heat and UfCB + O_3_ exposure and that its deficiency alters the heart’s adaptive responses.

## 2. Materials and Methods

### 2.1. Exposure System and Aerosol Characterization

We previously described the whole-body inhalation exposure system [43,44,45]. Briefly, bulk CB (Printex^®^ 90, gift from Evonik, Frankfurt, Germany) was aerosolized using an acoustical generator (HPAG, IEStechno, Morgantown, WV, USA), and a Venturi pump (JS-60 M, Vaccon, Medway, MA, USA) was used to further deagglomerate it. A corona discharge device (HTU500AC, Ozone Solutions, Hull, IA, USA) was used to generate O_3_. Once generated, the UfCB + O_3_ aerosol was delivered into a custom stainless-steel chamber (Cube 150, IEStechno, Morgantown, WV, USA) where the mice were individually housed. A light scattering device (pDr-1500, Thermo Environmental Instruments Inc, Franklin, MA, USA) and a gas monitor (Model 202, 2B Technologies, Inc., Boulder, CO, USA) were used to monitor the aerosol mass concentration and the O_3_ levels, respectively. The UfCB + O_3_ exposure was successfully combined with HT because the exposure chamber’s insulated walls and CP-200HT, 200 W thermoelectric heat/cool plate, (TE technologies, Traverse, MI, USA) enabled it. Moreover, a custom heat exchanger and a fan assisted in homogeneous heat distribution inside the chamber. Automated feedback loops were used to monitor UfCB (2.5 mg/m^3^) and O_3_ (1 ppm) levels and ensure stable aerosols during the exposure period. Real-time monitoring of temperature and humidity (HMT330, Vaisala, Helsinki, Finland) was performed, and relative humidity was maintained at 40% level. Gravimetric measurements were also conducted on the aerosol, and the results of these measurements were used to continually calibrate the pDR-1500. Particle samples were collected periodically from the exposure chamber to analyze the particle size distribution. The particle size distribution of UfCB + O_3_ (2.5 mg/m^3^ + 1 ppm) aerosol was measured with or without HT using different particle sizers: (1) an electrical low-pressure impactor (ELPI+, Dakati, Tempera, Finland), (2) an aerosol particle sizer (APS 3321, TSI Inc Shoreview, MN, USA), (3) a scanning mobility particle sizer (SMPS 3938, TSI Inc. Shoreview, MN, USA) and (4) Micro-Orifice Uniform Deposit Impactor (Moudi 115R, MSP Corp, Shoreview, MN, USA). The elemental composition of particle surfaces was analyzed using X-Ray Photoelectron Spectroscopy (XPS) (Physical Electronics PHI 5000 VersaProbe XPS/UPS) and reported previously [43,44]. Particle number concentrations were measured using the condensation particle counter (CPC: TSI Inc., Shoreview, MN, USA). Particles were collected onto 25 mm diameter, 0.2 mm pore size track-etched polycarbonate filters (Sterlitech Corporation, VA, USA) by drawing air from the exposure chamber at 1 LPM for ~45 s using a pre-calibrated sampling pump with a modified NIOSH 7300 method [46]. Nitrogen Dioxide (NO_2_) concentration was monitored using electrochemical sensors.

### 2.2. Murine Model and Exposures

**Figure 1:**
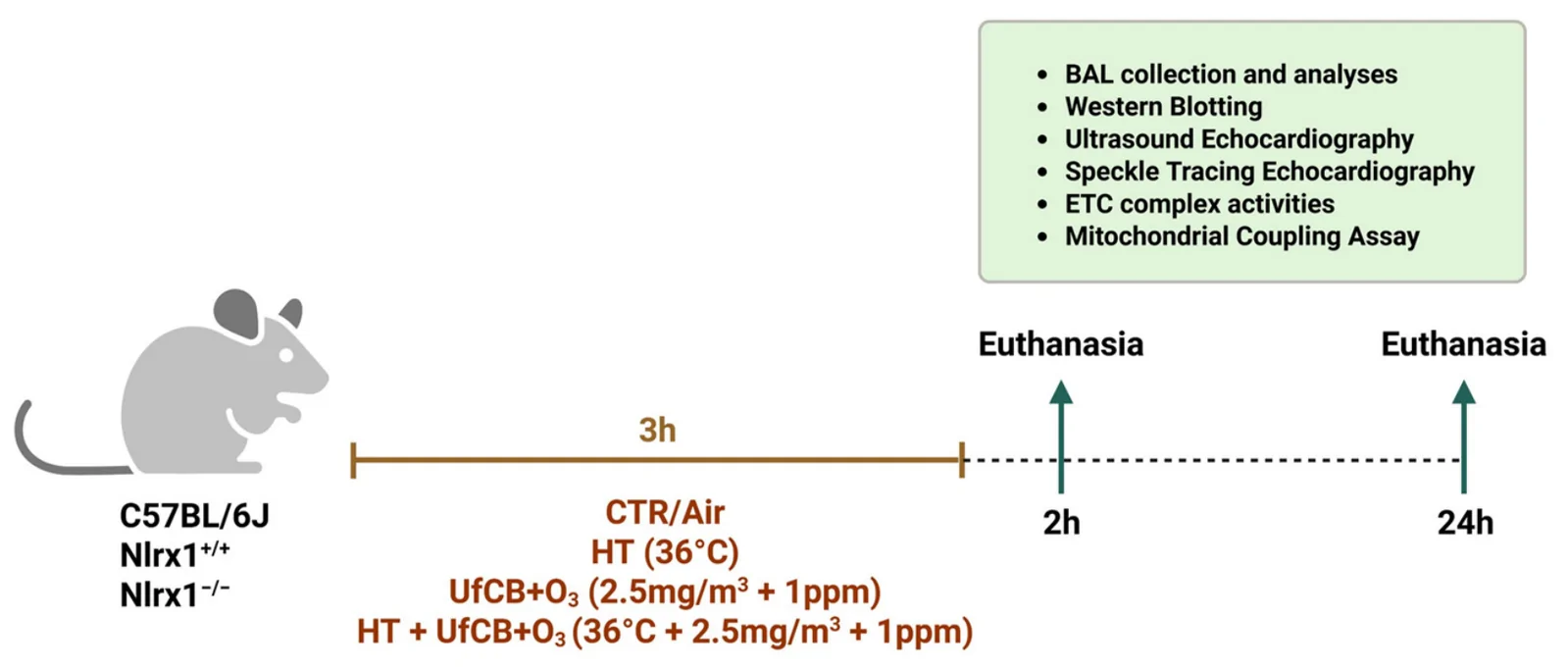
Experimental design. Female C57BL/6J *Nlrx1^+/+^* and *Nlrx1^−/−^* mice were randomized into different groups: (1) CTR/Air, (2) HT (36 °C), (3) UfCB + O_3_ (2.5 mg/m^3^ + 1 ppm), and (4) HT + UfCB + O_3_ (36 °C) + (2.5 mg/m^3^ + 1 ppm), exposed for 3 h and euthanized at 2 h or 24 h post-exposure. For clarity and conciseness in the text and figures, the groups are labeled as (1) CTR/Air, (2) HT, (3) UfCB + O_3_, and (4) HT + UfCB + O_3_, with 2 h or 24 h indicating the respective time points.

The experiments were conducted using female C57BL/6J mice at 8–10 weeks of age purchased from Jackson Laboratory (Bar Harbor, ME, USA). All animals were first acclimatized at the West Virginia University Animal Care Facility before experimentation. Animals were maintained in a 12 h light/dark cycle and in social housing (five mice/cage), supplied chow and water ad libitum. The West Virginia University (WVU) Animal Care and Use Committee approved all the animal procedures performed for this study. The *Nlrx1^−/−^* mice were acquired through an MTA from the University of North Carolina (UNC) [32]. All animal exposures were performed at the WVU Center for Inhalation Toxicology (iTOX). Throughout the duration of the exposure (3 h), mice were single-housed in steel mesh cages, and particle concentration, aerosol levels, temperature, and humidity were monitored to ensure stable and constant levels in the exposure chamber. Mice were randomly divided into 4 groups: (1) CTR/Air, (2) HT (36 °C), (3) UfCB + O_3_ (CB 2.5 mg/m^3^ + O_3_ 1 ppm), (4) HT + UfCB + O_3_ (36 °C) + (CB 2.5 mg/m^3^ + O_3_ 1 ppm) and were exposed accordingly for 3 h. Animals exposed only to filtered air, with no additional environmental exposure, were classified as control animals and labeled as the CTR/Air group. Animals were euthanized either 2 h or 24 h after the exposure. An intraperitoneal injection of Fatal Plus^®^ (pentobarbital sodium, 250 mg/kg, Vortech Pharmaceuticals LTD, Dearborn, MI, USA) was used to perform euthanasia. The experimental design is shown in Figure 1. Animals were housed at 22 °C, and the thermoneutral zone (the temperature at which animals do not need to actively expend energy to maintain core body temperature) for laboratory mice is between 26 °C and 34 °C. At 36 °C, animals must actively expend energy to dissipate heat, so this temperature induces heat stress. This is also a highly relevant environmental temperature, as 36 °C/96.8 °F is routinely observed during the summer months, even in the Western Hemisphere, including Europe and the USA. When humidity (40%) in the chamber is accounted for, this is an “extreme caution” heat index (feels like 101 °F) according to the United States National Weather Service (NWS).

### 2.3. Bronchoalveolar Lavage Fluid Collection and Analyses

Bronchoalveolar lavage (BAL) fluid was collected and quantified, as previously described [43,44,47,48]. Briefly, after euthanasia, half-lung lavage was performed on the right lung lobes. A total of 0.5 mL of cold PBS was instilled into the right lobes through the trachea and collected. The process was repeated three times to collect approximately 1.5 mL of BAL fluid. An automated counter, Countess II (Invitrogen, Waltham, MA, USA), was used to determine the total cell number in BAL fluid. Then, BAL fluid was centrifuged at 300× *g* for 5 min at 4 °C; the supernatant was collected. The total protein contents (TPA) in the BAL fluid were quantified using the Pierce BCA Assay Kit (Thermo Fisher Scientific) as per the manufacturer’s instructions. The cell pellet was resuspended in PBS, and slides were prepared by Cytospin (Thermo Fisher Scientific, Waltham, MA, USA). Slides were stained with Hema 3 (Fisher, Scientific, Pittsburgh, PA, USA) for the differential count analysis. Uptake of UfCB particles in the lavage macrophages was assessed in terms of % CB (black aggregates)-positive lavage macrophage cells. As previously described, a minimum of 500 cells were analyzed to assess the percentage of CB-positive macrophages. For differential analysis, 10,000 lavage cells were centrifuged onto slides using a Cytospin instrument (Thermo Fisher Scientific) and subsequently stained with Hema 3 (Fisher Scientific) [48]. The cell differential count data are presented as mean ± SD. The results at 2 h post-exposure and are *n* = 4–5 CTR/Air, *n* = 5–6 HT 2 h, *n* = 4–5 UfCB + O_3_ 2 h, and *n* = 4 HT + UfCB + O_3_. The results at 24 h post-exposures are *n* = 5CTR/Air, *n* = 4–9 HT 24 h, *n* = 4–6 UfCB + O_3_, and *n* = 4 HT + UfCB + O_3_.

### 2.4. Western Blotting

NLRX1 protein levels in lung tissues were evaluated by immunoblotting. Briefly, the total proteins were collected from grinded lung tissue using a mixture of RIPA buffer (Thermo Fisher Scientific, Waltham, MA, USA) supplemented with protease and phosphatase inhibitor cocktail (Sigma-Aldrich, St. Louis, MO, USA). The total proteins were quantified using the BCA Protein Assay Kit (Thermo Fisher Scientific, Waltham, MA, USA). NuPAGE™ Bis-Tris Mini Protein Gels, 4–12% was used for running. Then proteins were transferred to a PVDF membrane, blocked using 5% milk and then incubated overnight at 4 °C with 1:1000 dilution of NLRX1 antibody, (Proteintech, Rosemont, IL, USA, Cat. No. 17215-1-AP). The membrane was extensively washed after incubation, and 1:3000 dilution of anti-HRP-conjugated secondary antibody (anti-rabbit HRP, Cat No. 7074, Cell Signaling, Danvers, MA, USA) was applied for 1 h at room temperature. β-actin [(C4) HRP 1:20,000, (Santa Cruz Biotechnology, Dallas, TX, USA, Cat. No. sc-47778)] was used as an internal control. The bands were visualized using the Cytiva Amersham™ ECL kit (Marlborough, MA, USA) and detected with an Amersham Biosciences Imager 680 (GE Healthcare, Chicago, IL, USA). The relative protein levels were analyzed using ImageJ version 1.54i (National Institutes of Health, Bethesda, MD, USA). The results are expressed in NLRX1 expression post 2 h from exposure as the mean ± SD of *n* = 3 mice per each experimental group.

### 2.5. Cardiac Ultrasound Echocardiography

Cardiac function was assessed either 2 or 24 h after exposure using ultrasound Doppler echocardiography (Vevo F2 High-Frequency Ultrasound Echocardiography). Every mouse was anesthetized in an induction chamber with inhalant isoflurane at 5% in 100% oxygen. When fully anesthetized, the mouse was transferred to the imaging platform in a supine position and maintained at 2% isoflurane for the duration of the imaging. The scanning platform is equipped with a heated mouse-sized platform and integrated with electrodes for physiological monitoring, keeping the animal at body temperature and monitoring its heart rate (HR) and respiratory rate during the exams. The images were captured using a UHF57x transducer (57–25 MHz) (FUJIFILM VisualSonics, Toronto, ON, Canada) with a scanning depth of up to 10 mm and with a frame rate of 385–499 fps. The US transducer was first placed on the left side of the animal’s chest, with the notch oriented towards its right shoulder, identifying the parasternal long axis of the heart. A 90° clockwise rotation from this position generates a short axis view. Conventional echocardiographic assessments along the short axis were made from left ventricle (LV) M-mode images. The obtained measurements of stroke volume (SV), cardiac output (CO), ejection fraction percentage (EF%) and fractional shortening percentage (FS%) were calculated over 3 consecutive cardiac cycles and then averaged. All the ultrasound echocardiography measurements are presented as mean ± SD. The results are *n* = 6–7 of CTR/Air, *n* = 3–8 of HT 2 h, and *n* = 5 for HT 24 h mice for the experimental groups. The results of UfCB + O_3_ and HT single and co-exposures cardiac effects are *n* = 6 of CTR/Air, *n* = 4–8 of HT 2 h, *n* = 4 of UfCB + O_3_ 2 h, and *n* = 4 of HT + UfCB + O_3_ 2 h mice for each experimental group.

### 2.6. Speckle Tracking Echocardiography

Strain assessments were performed using parasternal short and long axes B-mode video loops analyzed with Vevo Lab v. 3.1.1 software (VevoStrain, Visual Sonics, Toronto, ON, Canada) [49]. The measurements were run using speckle tracking software by tracing the endocardial and epicardial walls of LV throughout the 3 cardiac cycles. The LV in the parasternal short axis was segmented into six regions of interest: (7) mid anterior, (8) mid anteroseptal, (9) mid inferoseptal, (10) mid inferior, (11) mid inferolateral and (12) mid anterolateral. Additionally, the LV in the parasternal long axis was segmented into seven regions of interest: (2) basal anteroseptal, (5) basal inferolateral, (8) mid anteroseptal, (11) mid inferolateral, (14) apical septal, (16) apical lateral and (17) apex. Segmental data were averaged within each experimental group and analyzed by heart wall layers (endocardium, myocardium, and epicardium). The results were expressed in longitudinal and circumferential time-to-peak percentage of strain (TTP%) using Visual Sonics VevoStrain 2.2.0 software (Toronto, ON, Canada) and employing a speckle tracking algorithm. Data are presented as mean ± SD. The radial plot results are *n* = 6–7 of CTR/Air, *n* = 4–8 of HT 2 h, *n* = 3–4 of UfCB + O_3_ 2 h, and *n* = 4 of HT + UfCB + O_3_ 2 h mice. The cumulative strain plot reports longitudinal and circumferential TTP% as the mean values of all three heart layers.

### 2.7. Mitochondrial Isolations

Heart tissue mitochondria were isolated from both *Nlrx1^+/+^* and *Nlrx1^−/−^* mice using differential centrifugation, as previously detailed [43,50,51]. Mitochondria were either immediately used for investigations or stored at −80 °C in KME buffer (100 mM KCL, 50 mM MOPS, and 500 µM at pH 7.4 in water).

### 2.8. Electron Transport Chain (ETC) Complex Activities

After 2 h and 24 h post-exposure, mice hearts from all experimental groups (CTR/Air, HT, UfCB + O_3_, HT + UfCB + O_3_) were collected, the heart tissue mitochondria were isolated, and the activities of ETC complexes (I, II, III, IV, and V) were measured spectrophotometrically, as previously described [50,51,52,53,54]. Briefly, NADH oxidation at 340 nm was measured to depict complex I activity, reduction in cytochrome c at 550 nm in the presence of reduced decylubiquinone (50 mM) was measured to define complex II activity, and the oxidation of cytochrome c at 550 nm was evaluated to define complex IV activity. Protein content was normalized using the Bradford method, with bovine serum albumin protein assay standards. The results were expressed as activities in nanomoles of substrate consumed per minute per milligram of protein. ATP synthase activity was measured as oligomycin-sensitive ATPase activity using an assay coupled with pyruvate kinase, as previously described [55,56,57,58]. Protein content was assessed as above, with final values expressed as nanomoles consumed per minute per milligram of protein, which was equal to the nanomoles of NADH oxidized per minute per milligram of protein. Data are presented as mean ± SD. The ETC results at 2 h post-exposure are *n* = 4 of CTR/Air, *n* = 4 of HT 2 h, *n* = 4–5 of UfCB + O_3_ 2 h, and *n* = 5 of HT + UfCB + O_3_ 2 h mice. Each mouse sample was run in experimental triplicate for complex activity assays. The ETC results at 24 h post-exposure are *n* = 3 animals per experimental group.

### 2.9. Mitochondrial Coupling Assay

The Seahorse XFe96 Electron Flow Assay was carried out, as previously reported [43,50]. In brief, the XFe96 analyzer was equilibrated at 37 °C overnight prior to the assay. Cardiac mitochondria were isolated from tissues harvested at 2 and 24 h after HT exposure and plated at a concentration of 2.5 µg per well in the XFe96 assay plate. Mitochondria were seeded in a total volume of 50 µL containing 1× mitochondrial assay solution (MAS), which consisted of 70 mM sucrose, 220 mM mannitol, 10 mM KH_2_PO_4_, 5 mM MgCl_2_, 2 mM HEPES, 1 mM EGTA, and 0.2% fatty acid–free BSA, and was supplemented with 10 mM pyruvate, 2 mM malate, and 4 µM of carbonyl cyanide 4-(trifluoromethoxy)phenylhydrazone (FCCP). Following centrifugation of the XF plate (2000× *g* for 20 min), additional MAS was added to each well to reach a final volume of 175 µL. The plate was then incubated for 10 min at 37 °C in a non-CO_2_ incubator. The XF sensor cartridge was prepared for compound injection, with 25 µL loaded into each port (A–D). Port A was loaded with rotenone (20 µM), Port B with succinate (100 mM), Port C with antimycin A (40 µM), and Port D with ascorbate (10 mM) and TMPD (1 mM). The sensor cartridge was calibrated using the instrument’s automated calibration protocol, as previously described [59]. The assay was then performed using a modified injection sequence consisting of four compounds instead of the standard three: ADP, oligomycin, FCCP, and rotenone/antimycin A. Data are presented as mean ± SD. The results are *n* = 3–4 of CTR/Air, *n* = 4–5 of HT 2 h, and *n* = 3–4 of HT 24 h for each experimental group.

### 2.10. Statistical Analyses

Data are presented as mean ± standard deviation of the mean (SD) with a total of 4–9 animals per group. Significant differences between groups were identified using analysis of variance (one-way or two-way, as dictated by the experimental design), followed by Tukey’s post hoc test for multiple comparisons. Specifically, one-way ANOVA was used solely for the WB analyses, while the remaining data were analyzed with two-way ANOVA, considering genotype and exposures as factors, along with their interaction. For the ETC activity and Seahorse experiments, we analyzed experimental triplicates for each mouse and presented the mean in the graphs. All statistical analyses were performed using GraphPad Prism Software Version 10 for Windows (GraphPad Software, La Jolla, CA, USA). A two-tailed *p*-value of less than 0.05 was considered statistically significant.

## 3. Results

### 3.1. Heart Function Changes After HT Exposure

**Figure 2:**
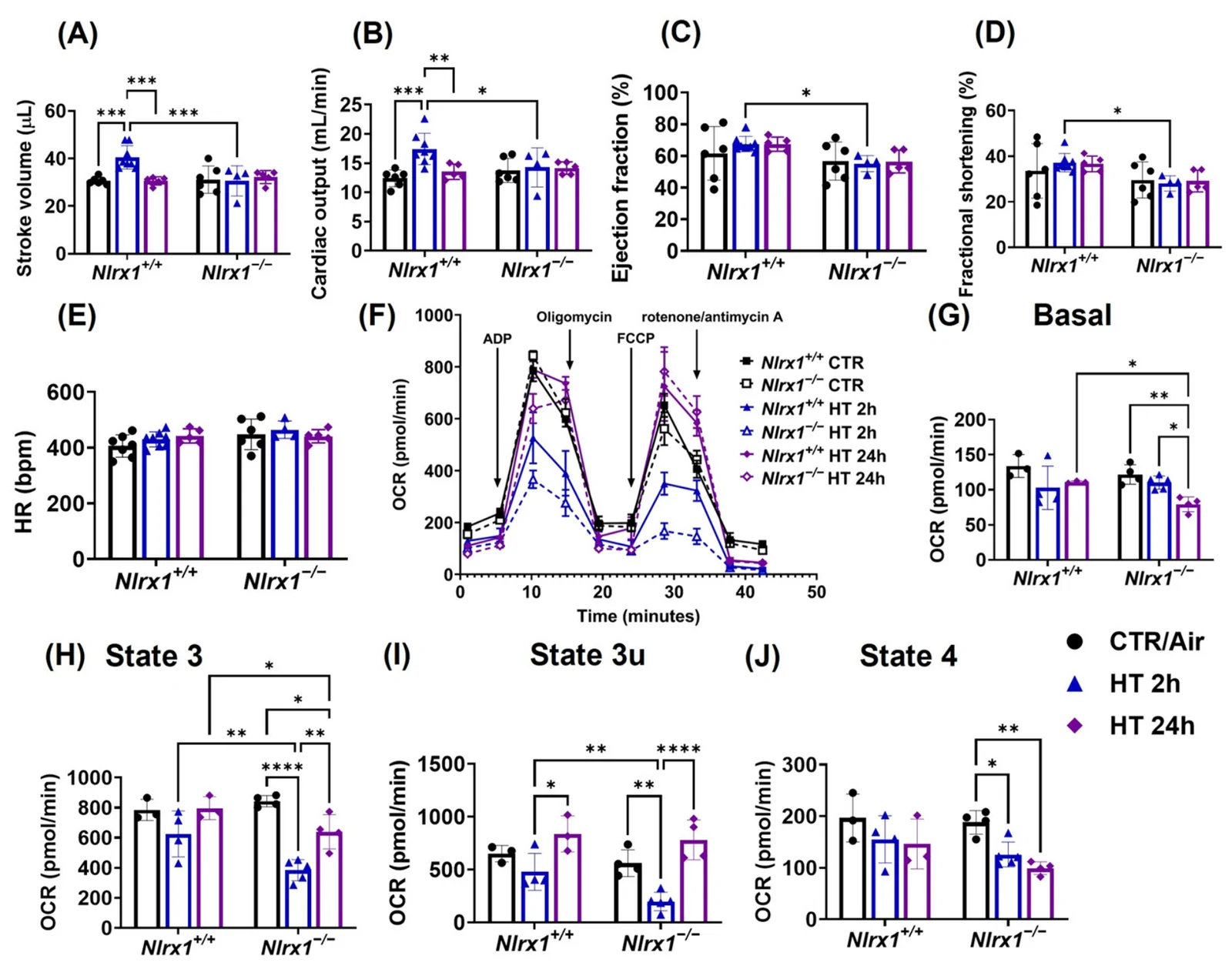
Ultrasound cardiac function assessment 2 h post-HT exposure. Ultrasound echocardiography measurements of stroke volume (SV), cardiac output (CO), ejection fraction percentage (EF%), fractional shortening percentage (FS%) and heart rate (HR) of Nlrx1^+/+^ mice and Nlrx1^−/−^ mice (**A**–**E**) after CTR/Air, HT 2 h, and HT 24 h exposures. Data are presented as mean ± SD of *n* = 4–8 mice per group. Data were analyzed by two-way ANOVA followed by Tukey’s post hoc test for multiple comparisons. (**F**) Seahorse XFe96 measurement for oxygen consumption rate (OCR) at pmol/min of both genotypes 2 h and 24 h post CTR/Air, and HT exposures with (**G**) Basal (**H**) State 3 (**I**) State 3u (**J**) State 4 respiration OCR values. Data are presented as mean ± SD of *n* = 3–5 mice per group and analyzed with two-way ANOVA followed by Tukey’s post hoc test. * *p* < 0.05, ** *p* < 0.01, *** *p* < 0.001, **** *p* < 0.0001.

To evaluate the impact of heat stress on cardiac function, M-mode echocardiographic images were acquired at 2 and 24 h following HT exposure. *Nlrx1^+/+^* mice exhibited increases in stroke volume (SV) and cardiac output (CO) at 2 h, with both measures returning to baseline 24 h post-exposure (Figure 2A,B). However, no significant change in *Nlrx1^+/+^* EF% and FS% was detected after HT exposure (Figure 2C,D). In contrast, *Nlrx1^−/−^* mice displayed no significant alterations compared to same-genotype CTR/Air controls but demonstrated lower overall response compared to heat-exposed *Nlrx1^+/+^* mice (Figure 2A–D). In both genotypes HT exposure did not significantly modulate HR (Figure 2E). To investigate the apparent lack of response of *Nlrx1^−/−^* hearts to heat exposure, mitochondrial function was assessed in isolated heart mitochondria. OCR decreased in both genotypes 2 h post-HT, with a greater reduction in *Nlrx1^−/−^* mice; by 24 h, *Nlrx1^+/+^* mice largely recovered OCR levels, whereas Nlrx1^−/−^ hearts showed only partial recovery In contrast, *Nlrx1^−/−^* mice displayed no significant alterations compared to same-genotype CTR/Air controls but demonstrated lower overall response compared to heat-exposed *Nlrx1^+/+^* mice (Figure 2A–D). In both genotypes HT exposure did not significantly modulate HR (Figure 2E). To investigate the apparent lack of response of *Nlrx1^−/−^* hearts to heat exposure, mitochondrial function was assessed in isolated heart mitochondria. OCR decreased in both genotypes 2 h post-HT, with a greater reduction in *Nlrx1^−/−^* mice; by 24 h, *Nlrx1^+/+^* mice largely recovered OCR levels, whereas Nlrx1^−/−^ hearts showed only partial recovery (Figure 2F). Basal mitochondrial OCR in *Nlrx1^+/+^* hearts remained unchanged following exposure, whereas mitochondria from *Nlrx1^−/−^* hearts demonstrated a significant reduction at 24 h compared with both HT 2 h and control conditions, as well as HT 24 h *Nlrx1^+/+^* hearts (Figure 2G). Moreover, *Nlrx1^−/−^* mice heart mitochondria displayed a significant reduction in ADP-driven state 3 respiration in FCCP-induced maximal uncoupled-stimulated state 3u and in state 4 respiration at 2 h post-HT exposure (Figure 2H–J). Importantly, state 3 OCR in *Nlrx1^−/−^* hearts remained significantly lower than the corresponding *Nlrx1^+/+^* levels at 24 h (Figure 2H). State 4 respiration in *Nlrx1^−/−^* heart mitochondria remained significantly low even at 24 h post-exposure (Figure 2J).

### 3.2. Aerosol Characteristics

**Figure 3:**
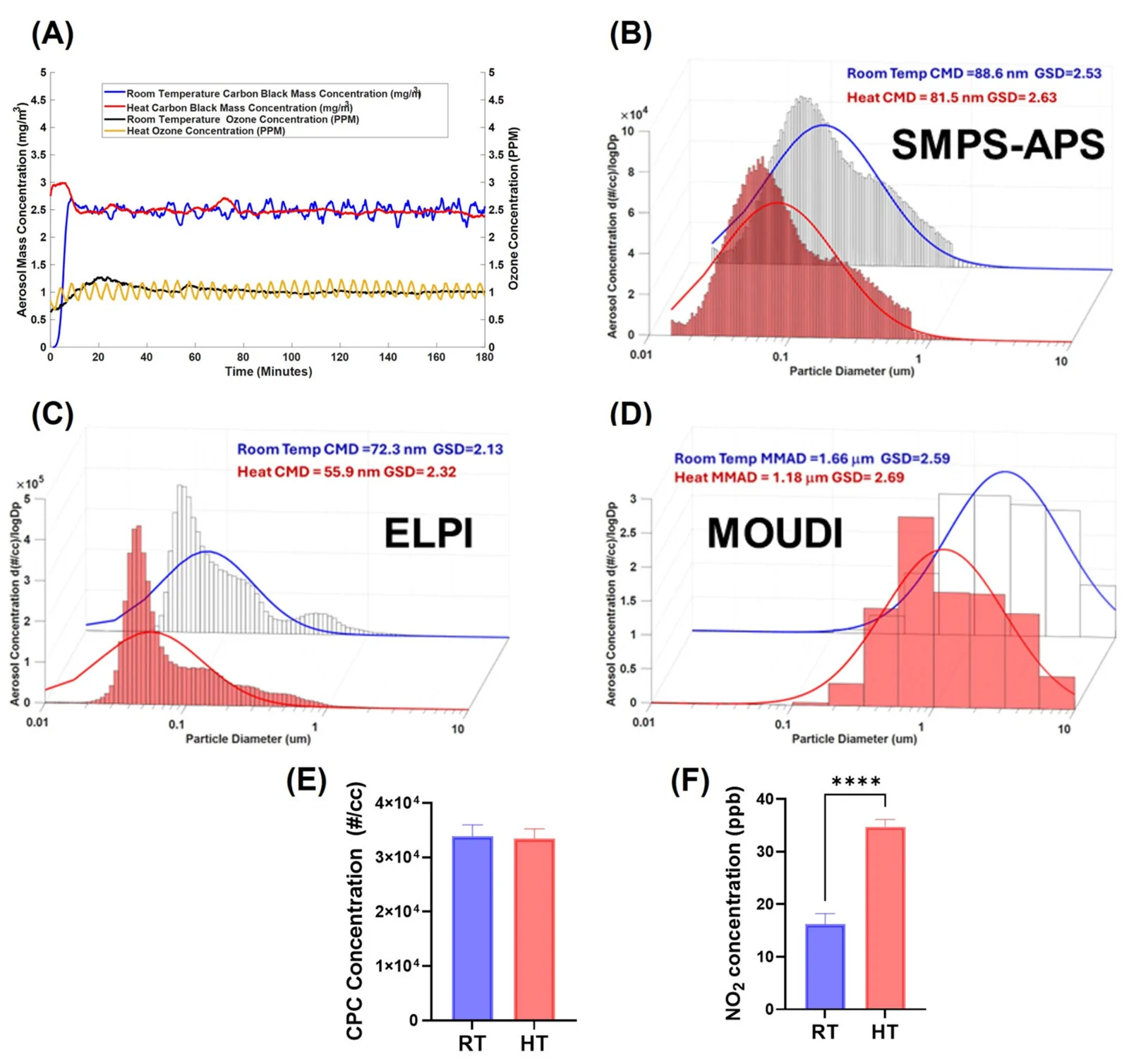
Aerosol characteristics. (**A**) Real-time monitoring of UfCB and O_3_ concentrations in the exposure chamber during the exposure period. Representative aerosol size distribution profiles obtained using (**B**) SMPS/APS (scanning mobility particle sizer/aerodynamic particle sizer), (**C**) ELPI+ (electrical low-pressure impactor), and (**D**) MOUDI (Micro-Orifice Uniform Deposit Impactor). (**E**) Particle concentrations by CPC (condensation particle counter), and (**F**) NO_2_ concentration with or without heat. Data are presented as mean ± SD of *n* = 4 exposures and analyzed by *t*-test. **** *p* < 0.0001.

The real-time monitoring of CB and O_3_ levels with and without HT and the gravimetric measurements showed a stable aerosol over the exposure period of 3 h (Figure 3A). Concurrent SMPS/APS measurements indicated no impact of HT on aerosol size distribution, with most particles in the nano/ultrafine size range (RT count median diameter of 88.6 nm and geometric standard deviation of 2.53; HT count median diameter of 81.5 nm with a geometric standard deviation of 2.63) (Figure 3B). Charge-based particle size measurements conducted with the ELPI+ resulted in a median diameter of 72.3 nm with a geometric standard deviation of 2.13 for RT aerosols and a count median diameter of 55.9 nm and a geometric standard deviation of 2.32 for HT aerosols (Figure 3C). Mass-based Moudi measurements showed a mass median aerodynamic diameter of 1.66 µm with a geometric standard deviation of 2.59 for RT aerosols and 1.18 µm and a geometric standard deviation of 2.69 for HT aerosol (Figure 3D). During exposure, the CPC estimated concentrations were 3.39 × 10^4^ ± 2090/cc at RT + UfCB + O_3_ and 3.35 × 10^4^ ± 1750/cc in HT + UfCB + O_3_ conditions (Figure 3E); NO_2_ concentration was significantly increased at HT compared to that at RT (16.18 ± 2.04 ppb at RT and 34.70 ± 1.41 ppb at HT (Figure 3F).

### 3.3. HT + UfCB + O_3_ Co-Exposure Induces Greater Lung Inflammation at 2 h Post-Exposure in Nlrx1^−/−^ Mice

**Figure 4:**
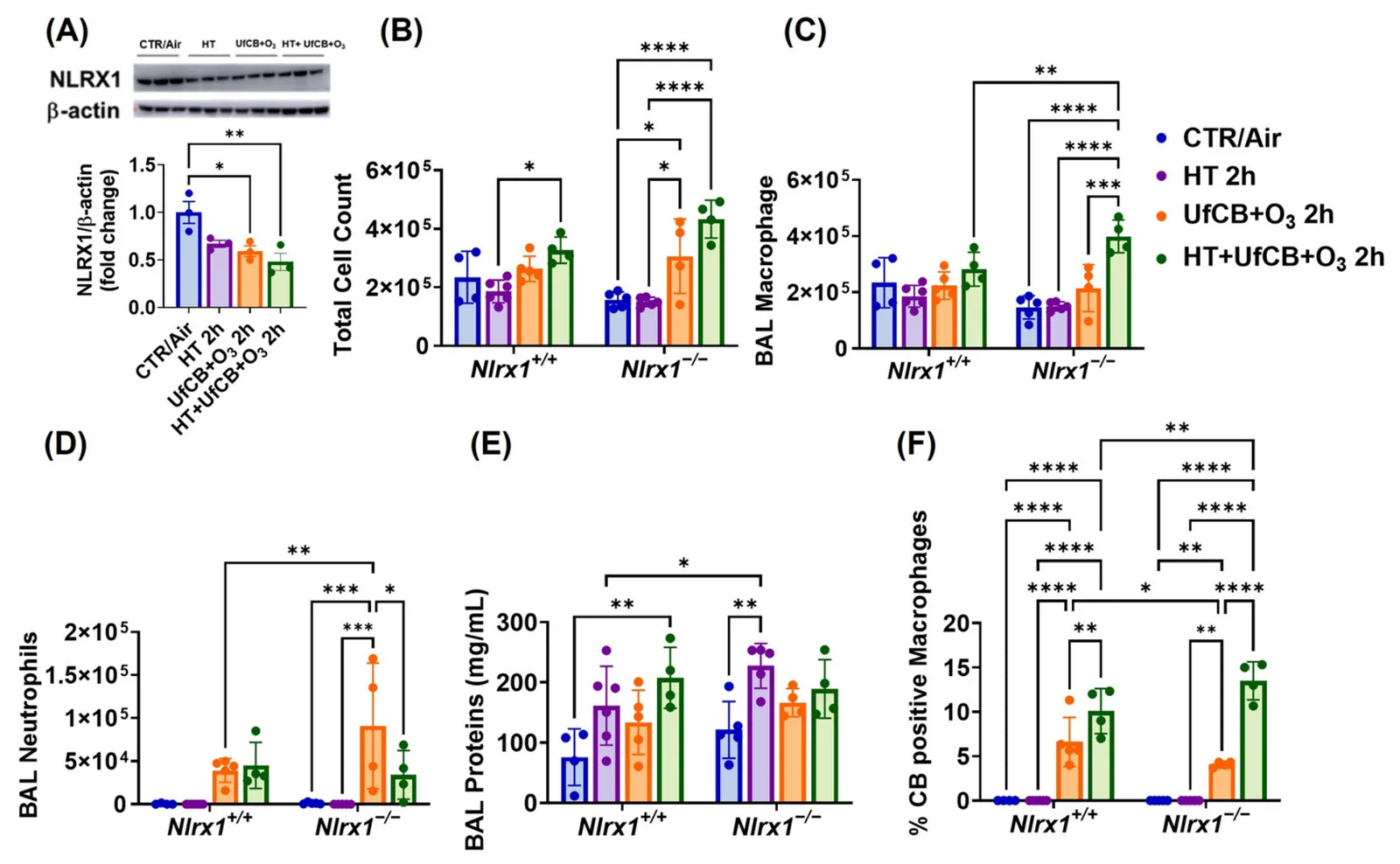
Lung inflammation assessment at 2 h post-exposure. (**A**) Representative Western blot image and graph showing lung NLRX1 expression after CTR/Air, HT, UfCB + O_3_ or HT + UfCB + O_3_ exposures (*n* = 3 mice per group). (**B**) Bronchoalveolar lavage total cells, (**C**) macrophages, (**D**) neutrophils, (**E**) total protein and (**F**) % CB-positive macrophages. Data are presented as mean ± SD of *n* = 4–6 mice per group and analyzed by two-way ANOVA followed by Tukey’s post hoc test. * *p* < 0.05, ** *p* < 0.01, *** *p* < 0.001, **** *p* < 0.0001.

Western blot analysis of lung lysates demonstrated significantly reduced NLRX1 expression at 2 h after UfCB + O_3_ and HT + UfCB + O_3_ exposures (Figure 4A). At 2 h post-HT-alone exposure, there was no increase in BAL cell counts in either genotype. Unlike *Nlrx1^+/+^* mice, *Nlrx1^−/−^* mice demonstrated increased BAL cell counts after UfCB + O_3_ and HT + UfCB + O_3_ exposure (Figure 4B). This increase in *Nlrx1^−/−^* mice BAL cell counts was primarily driven by increased numbers of macrophages and neutrophils (Figure 4C,D). The neutrophil numbers showed a modest increase in both genotypes; however, the *Nlrx1^−/−^* UfCB + O_3_ exposure group demonstrated significantly greater increase compared to the same group in *Nlrx1^+/+^* mice (Figure 4D). A trend of elevated BAL total protein levels was observed in all groups, indicating damage to the alveolar–capillary barrier, while a statistically significant increase was only observed in HT-exposed *Nlrx1^−/−^* mice. Furthermore, UfCB + O_3_ exposure increased the percentage of CB-positive macrophages in both genotypes. However, HT + UfCB + O_3_ co-exposure resulted in a significant increase in CB-positive macrophages in both *Nlrx1^+/+^* and *Nlrx1^−/−^* BAL compared to UfCB + O_3_ alone (Figure 4F).

### 3.4. Cardiac Functional Changes at 2 h Post-Exposure

**Figure 5:**
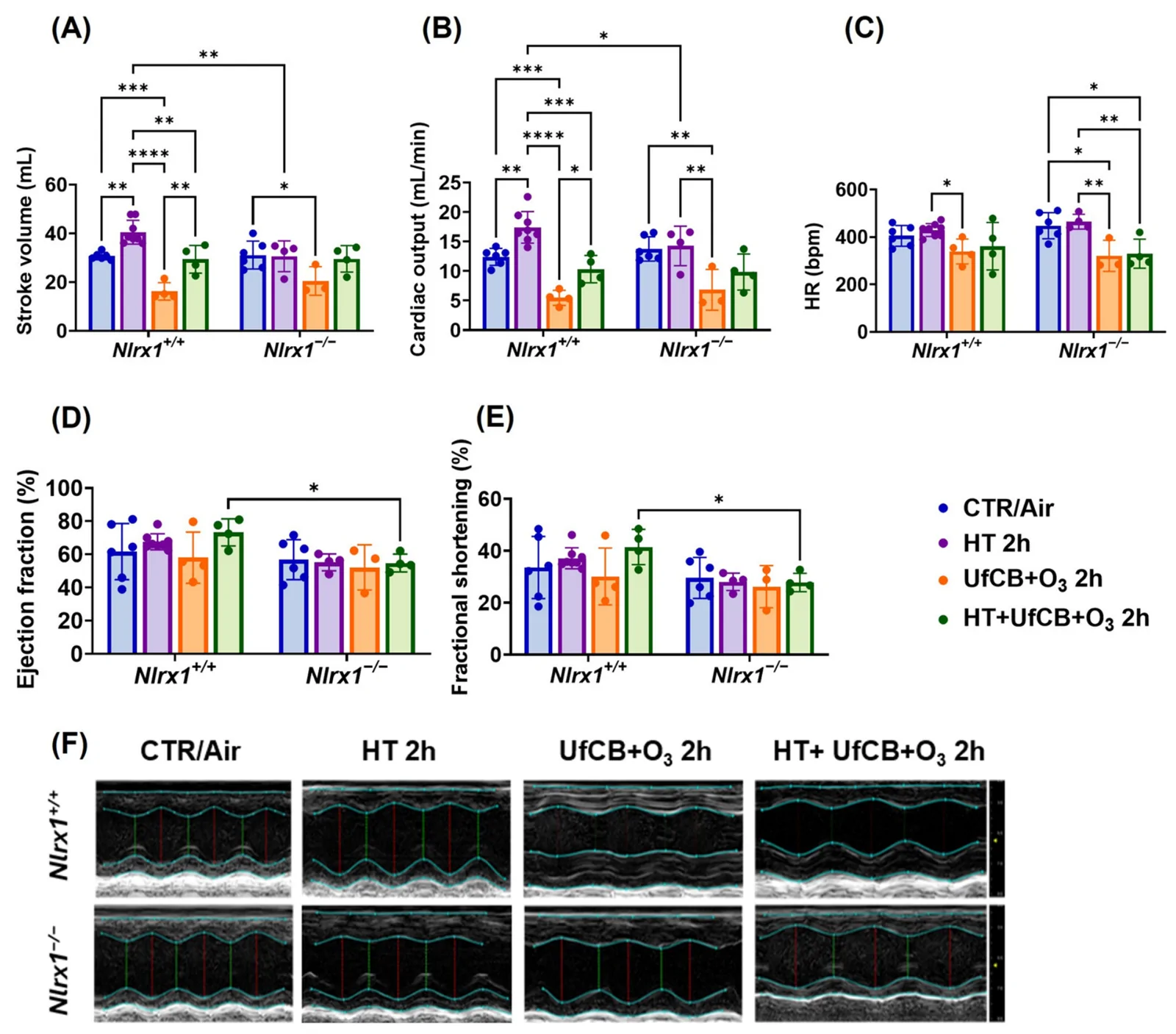
Ultrasound echocardiography assessment of cardiac function 2 h post-exposure. (**A**–**E**) Ultrasound echocardiography measurements of stroke volume (SV), cardiac output (CO), heart rate (HR), ejection fraction percentage (EF %) and fractional shortening percentage (FS %) of *Nlrx1^+/+^* mice and *Nlrx1^−/−^* mice after CTR/Air, HT, UfCB + O_3_, and HT + UfCB + O_3_ at 2 h post-exposures. Data are presented as mean ± SD of *n* = 3–8 mice per group and analyzed by two-way ANOVA followed by Tukey’s post hoc test. * *p* < 0.05, ** *p* < 0.01, *** *p* < 0.001, **** *p* < 0.0001. (**F**) Representative M-mode images of *Nlrx1^+/+^* and *Nlrx1^−/−^* mice exposed to CTR/Air, HT, UfCB + O_3_, and HT + UfCB + O_3_.

Subsequently, we assessed the effects of UfCB + O_3_ alone or combined with HT on cardiac function. *Nlrx1^+/+^* mice responded to HT by increasing SV and CO, while there was no significant change in these parameters in *Nlrx1^−/−^* mice (Figure 2A,B and Figure 5A,B). UfCB + O_3_ exposure negatively affected heart function in both genotypes, as indicated by a significant reduction in SV and CO and HR (Figure 5A–C). Exposure to either HT or UfCB + O_3_ alone did not affect EF% or FS% in either genotype (Figure 5D,E). However, HT + UfCB + O_3_ co-exposure was associated with a significant reduction in HR in *Nlrx1^−/−^* mice, whereas EF% and FS% were significantly lower in *Nlrx1^−/−^* mice compared to *Nlrx1^+/+^* mice (Figure 5D,E). The representative M-mode tracings are presented in Figure 5F.

**Figure 6:**
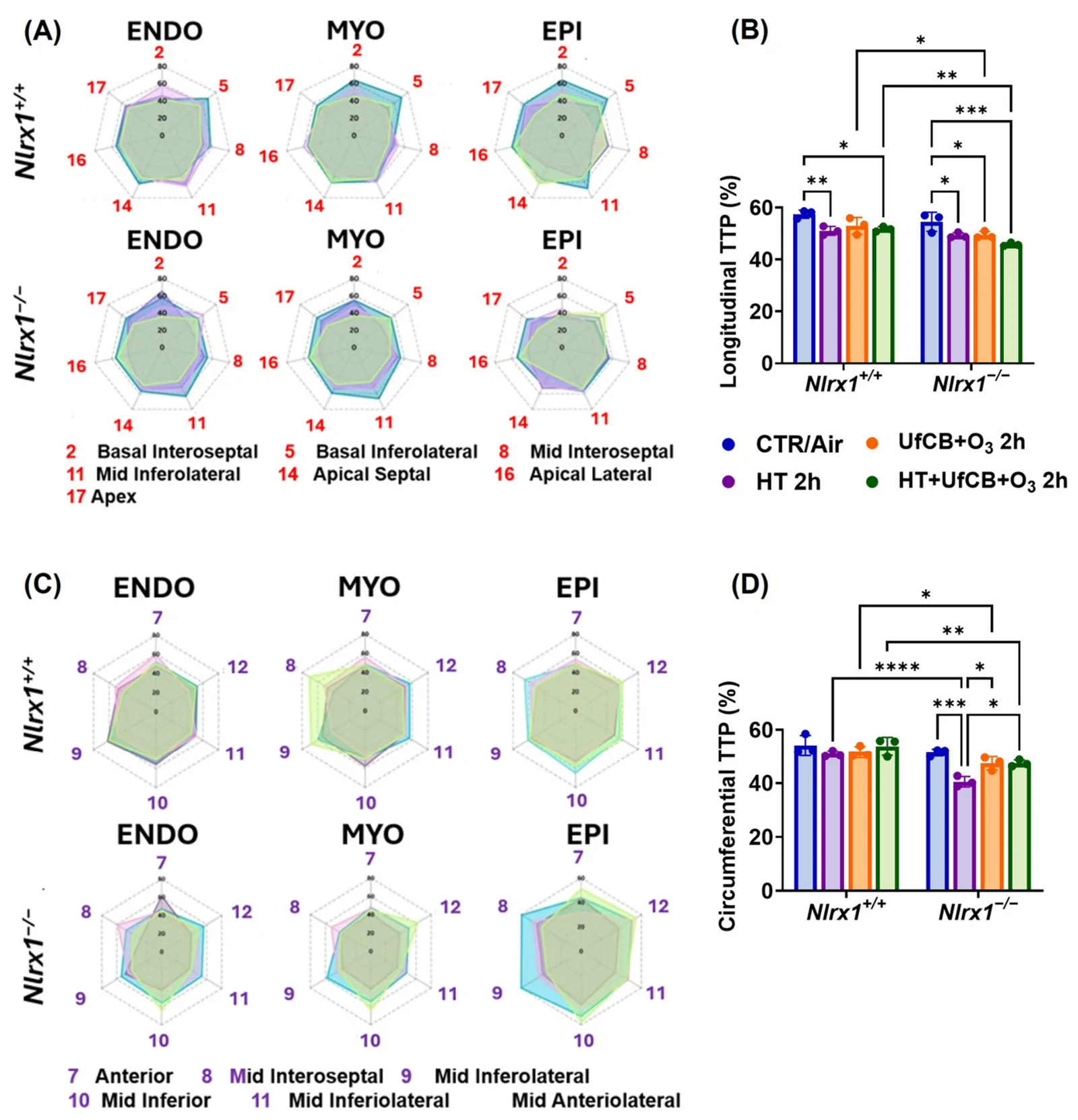
Speckle tracking strain echocardiography at 2 h post-exposure. Radial plots of regional strain, expressed as time-to-peak percentage (TTP%), along the (**A**) long axis and (**C**) short axis, and distinguished across the endocardium (ENDO), myocardium (MYO), and epicardium (EPI) layers of *Nlrx1^+/+^* and *Nlrx1^−/−^* hearts. Panels (**B**,**D**) show cumulative strain across all three layers, presented as longitudinal TTP% (long axis) and circumferential TTP% (short axis), respectively. Data are presented as mean ± SD for *n* = 3–8 animals per group for the radial plot and *n* = 3 heart layers for the cumulative strain plot. Data were analyzed by two-way ANOVA followed by Tukey’s post hoc test. * *p* < 0.05, ** *p* < 0.01, *** *p* < 0.001, **** *p* < 0.0001.

To further characterize potential contractile dysfunction, we quantified strain change via speckle tracking echocardiography, analyzing either regional (segmental) or cumulative strains. *Nlrx1^−/−^* hearts exhibited evidence of contractile dysfunction, characterized by increased mechanical dyssynchrony across longitudinal and circumferential heart mid-cavity segments. Layer-resolved analysis demonstrated distinct alterations in time to peak (TTP%) across the endocardium, myocardium, and epicardium, which were particularly pronounced following HT + UfCB + O_3_ co-exposure (Figure 6A,C). The cumulative longitudinal TTP % was significantly reduced by HT exposure in both genotypes. In contrast, UfCB + O_3_ exposure altered it only in *Nlrx1^−/−^* mice, resulting in a marked decline in TTP% relative to both the CTR/Air and *Nlrx1^+/+^* groups, indicating impaired contractile function. The cumulative longitudinal TTP% post-HT + UfCB + O_3_ exposure, a reflection of the overall deformation of endocardium and myocardium, confirmed a significantly reduced TTP% in *Nlrx1^−/−^* compared to *Nlrx1^+/+^* mice hearts (Figure 6B). Circumferential TTP% analysis further supported impaired heart contractility in *Nlrx1^−/−^* mice (Figure 6D). HT alone significantly reduced TTP% in *Nlrx1^−/−^* mice compared to the UfCB + O_3_ and HT + UfCB + O_3_ groups, as well as relative to the corresponding conditions in *Nlrx1^+/+^* mice (Figure 6D).

### 3.5. Heart Mitochondria ETC Complex Activities 2 h Post-Exposure

**Figure 7:**
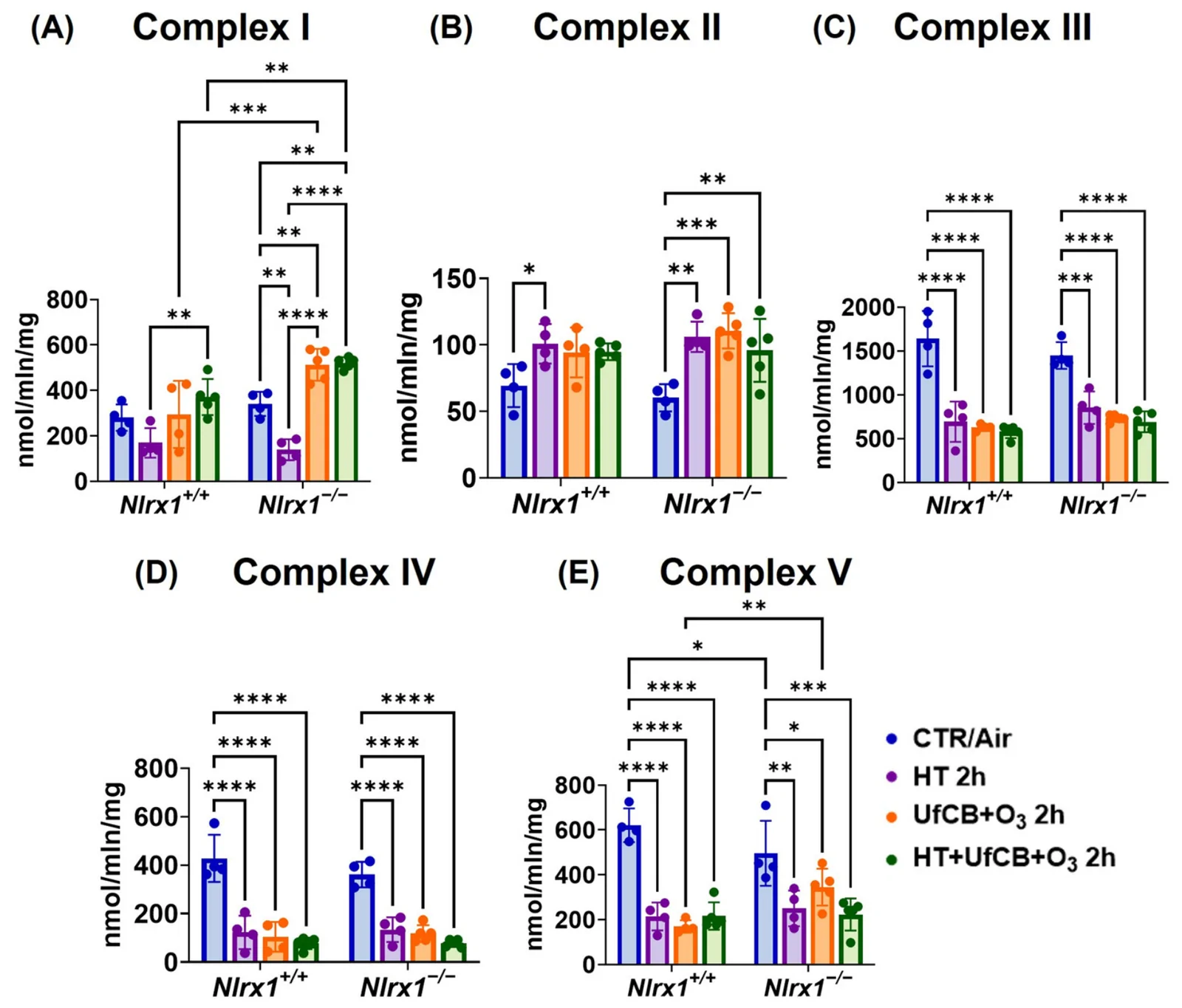
*ETC complex activities of isolated heart mitochondria* 2 h post-exposure. Mitochondrial ETC complex activities from *Nlrx1^+/+^* and *Nlrx1^−/−^* mouse hearts after CTR/Air, HT, UfCB + O_3_ and HT + UfCB + O_3_ exposures. The results are expressed as the amount of substrate consumed per minute per ng or mg of protein for (**A**) complex I, (**B**) complex II, (**C**) complex III, (**D**) complex IV, and (**E**) complex V (*n* = 4–5, per group). Data are presented as mean ± SD and analyzed with two-way ANOVA followed by Tukey’s post hoc. * *p* < 0.05, ** *p* < 0.01, *** *p* < 0.001, **** *p* < 0.0001.

We investigated mitochondria to elucidate the mechanistic basis of the observed cardiac functional changes by assessing mitochondrial electron transport chain (ETC) complex activities in isolated cardiac mitochondria 2 h after exposure. Complex I (CI) activity was significantly decreased by the HT exposure in *Nlrx1^−/−^* mice, and a trend of reduction was observed in *Nlrx1^+/+^* mice (Figure 7A). A significantly increased CI activity was quantified for UfCB + O_3_ and HT + UfCB + O_3_ exposure in *Nlrx1^+/+^* mice (Figure 7A). Complex II (CII) activity was significantly induced by HT irrespective of the genotypes but was only significantly induced by UfCB + O_3_ and HT + UfCB + O_3_ in *Nlrx1^−/−^* mice heart mitochondria (Figure 7B). Complex III (CIII), complex IV (CIV) and complex V (CV) activities were significantly decreased in both genotypes irrespective of the treatment (Figure 7C–E).

### 3.6. Lung Inflammation Induced by HT and UfCB + O_3_ Co-Exposure at 24 h

**Figure 8:**
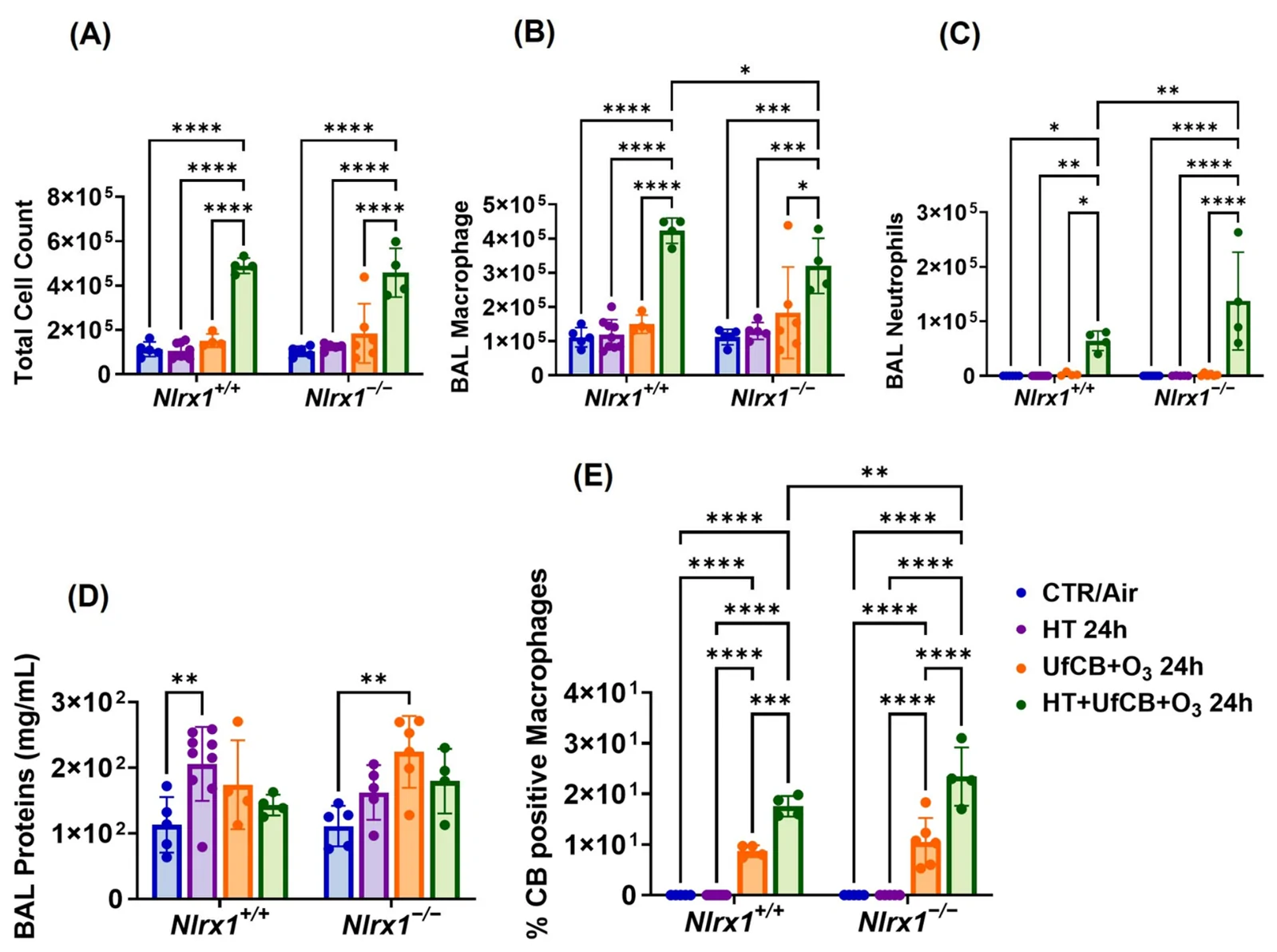
Lung inflammation assessment at 24 h post-exposure. (**A**) Bronchoalveolar lavage total cells, (**B**) macrophages, (**C**) neutrophils, (**D**) total protein, and (**E**) % CB-positive macrophages from *Nlrx1*^+/+^ and *Nlrx1*^−/−^ mice 24 h post CTR/Air, HT, UfCB + O_3_, or HT + UfCB + O_3_ exposure. Data are presented as mean ± SD of *n* = 4–9 mice per group and analyzed by two-way ANOVA followed by Tukey’s post hoc test. * *p* < 0.05, ** *p* < 0.01, *** *p* < 0.001, **** *p* < 0.0001.

In addition to the 2 h time point, BAL fluid was collected at 24 h post-exposure to assess pulmonary effects. At 24 h post-exposure, only HT + UfCB + O_3_ exposure induced a significantly greater total number of lavage immune cells (Figure 8A). This increase was due to a significant increase in macrophage and neutrophil counts after HT + UfCB + O_3_ exposure and was significantly greater in *Nlrx1^−/−^* mice compared to *Nlrx1^+/+^* mice (Figure 8B,C). BAL total protein levels were elevated in the HT-only group in *Nlrx1^+/+^* mice and UfCB + O_3_ in *Nlrx1^−/−^* mice, while other groups demonstrated an increasing trend (Figure 8D), indicating impaired lung barrier integrity under these conditions (Figure 8D). To assess macrophage uptake of UfCB particles, we measured the percentage of CB-positive macrophages. UfCB + O_3_ exposure increased CB-positive macrophages in both genotypes (Figure 8E). However, HT + UfCB + O_3_ co-exposure induced a greater increase in CB-positive macrophages in *Nlrx1^−/−^* mice compared to *Nlrx1^+/+^* mice (Figure 8E).

### 3.7. Heart Mitochondria ETC Complex Activities 24 h Post-Exposures

**Figure 9:**
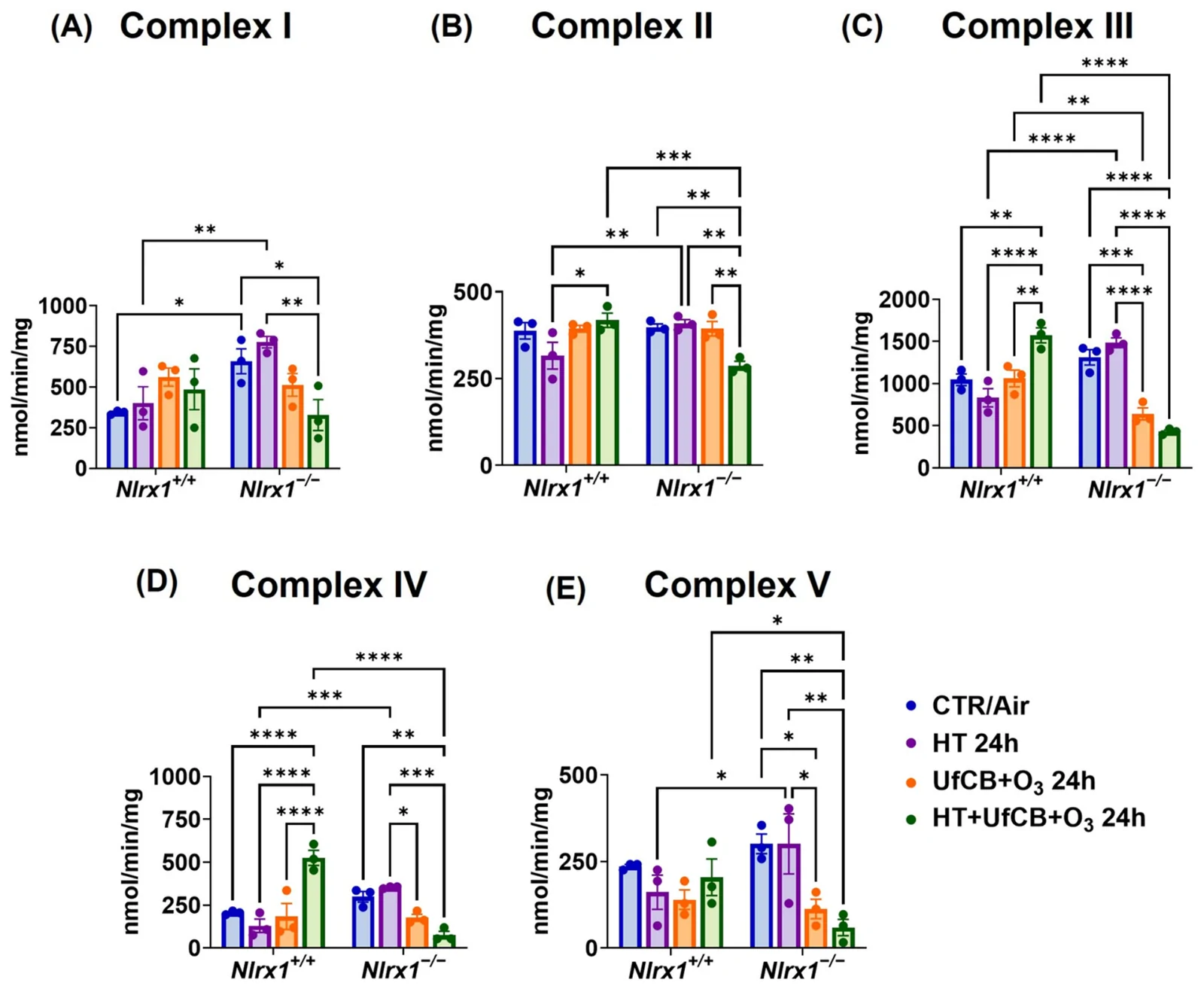
ETC complex activities of isolated heart mitochondria 24 h post-exposure. *Nlrx1^+/+^* and *Nlrx1^−/−^* heart mitochondria ETC complex activities were assessed 24 h post CTR/Air, HT, UfCB + O_3_ and HT + UfCB + O_3_ exposures. The results are expressed as the amount of substrate consumed per minute per ng or mg of protein for (**A**) complex I, (**B**) complex II, (**C**) complex III, (**D**) complex IV, and (**E**) complex V. Data are presented as mean ± SD from *n* = 3 mice per group. Each mouse heart mitochondria sample was analyzed as experimental triplicate in complex activity assays. Data are analyzed with two-way ANOVA followed by Tukey’s post hoc. * *p* < 0.05, ** *p* < 0.01, *** *p* < 0.001, **** *p* < 0.0001.

HT-induced changes in cardiac mitochondrial ETC complexes (CI–CV) activities returned to baseline at 24 h post-exposure. CI activity was significantly decreased only in the HT + UfCB + O_3_-exposed *Nlrx1^−/−^* mice cardiac mitochondria (Figure 9A). Similarly, CII activity was significantly reduced only by HT + UfCB + O_3_ in Nlrx1^−/−^ mice (Figure 9B). Interestingly, CIII and CIV activities were significantly increased by HT + UfCB + O_3_ in the Nlrx1^+/+^ mice, while it was significantly reduced by UfCB + O_3_ and HT + UfCB + O_3_ exposure in Nlrx1^−/−^ mice (Figure 9C,D). CV activity was significantly reduced by UfCB + O_3_ and HT + UfCB + O_3_ exposure only in Nlrx1^−/−^ mice (Figure 9E).

## 4. Discussion

In the present study, we investigated how co-exposure to HT and UfCB + O_3_ affects pulmonary inflammation and cardiac function. Our findings demonstrate that even a single acute, environmentally relevant exposure induces significant lung inflammation and alters cardiac physiological adaptation, accompanied by changes in mitochondrial function in the heart. These effects were particularly pronounced in the context of NLRX1 deficiency. Currently, the role of NLRX1 in HT-induced regulation of biological responses alone and in combination with UfCB + O_3_ is poorly understood.

Both air pollution and heat exposures in our study are environmentally relevant, as global PM_2.5_ concentrations frequently exceed the National Ambient Air Quality Standards (NAAQS), and nearly 90% of the global population is exposed to air quality above the World Health Organization (WHO) guidelines. In this study, mice were exposed to UfCB at 2.5 mg/m^3^ for 3 h; as already demonstrated, this exposure led to an alveolar-deposited dose comparable to that of a worker after 2 h of exposure at the current U.S. Occupational Safety and Health Administration (OSHA) permissible limit (3.5 mg/m^3^) or one-week cumulative exposure at the current 24 h PM_2.5_ standard (35 µg/m^3^). Due to anatomical differences and the sedentary nature of laboratory rodents, 1 ppm O_3_ exposure is used to achieve biological effects comparable to those observed in exercising humans exposed to 200–400 ppb O_3_ [60,61,62,63,64,65]. Finally, to model high heat exposure conditions concurrently with pollutant exposure, mice were maintained at 36 °C and 40% relative humidity (heat index 39.2 °C/102.5 °F), which triggers the National Weather Service “extreme caution” advisory but is not expected to cause mortality [66]. We performed these studies on combined CB and O_3_ exposures representing the mixed particle and gas nature of air pollution. Our previous work demonstrated that co-exposure to UfCB and O_3_ induces greater pulmonary toxicity, inflammation, lung function decline, and lung and gut microbiome alterations [44,48]. Furthermore, combined exposure to extreme heat and gaseous pollutants (ozone) has been associated with increased cardiovascular disease and mortality, highlighting the complex interactions between air pollution, climate extremes, and human health [15,24,67].

Our study identifies that NLRX1 deficiency alters the physiological cardiopulmonary responses to heat and air pollution co-exposure, demonstrating that loss of NLRX1 is linked to greater lung inflammation and reduced cardiac adaptive response. NLRX1 deficiency has already been shown to amplify inflammation, worsen mitochondrial and kidney injury, and regulate reactive oxygen species (ROS), autophagy, and apoptosis across multiple disease models [37,68,69,70]. Notably, oxidative stress and mitochondrial impairment drive chronic inflammation during respiratory disease [71,72] and lead to cardiac dysfunction and progression of various heart diseases [73,74,75,76,77]. When environmental temperatures rise beyond the thermoneutral zone, increasing core body temperature, heat-dissipating mechanisms are accompanied by critical autonomic and cardiovascular adjustments, including increased cardiac output and heightened sympathetic activity to redistribute blood from non-cutaneous regions. These thermoregulatory responses are essential for maintaining blood pressure and ensuring adequate organ perfusion [78,79,80]. In an HT exposure scenario, our results demonstrate the inability of *Nlrx1^−/−^* mice to undergo cardiac functional adaptation. Contrary to the expected increase in cardiac performance parameters reflecting enhanced pumping efficiency, *Nlrx1^−/−^* mice failed to exhibit significant cardiac adaptation over time in response to HT exposure. Notably, these mice showed significantly lower SV, CO, EF%, and FS% at 2 h post-exposure compared to *Nlrx1^+/+^* mice. These results suggest impaired systolic function, likely due to reduced myocardial contractility, leading to decreased blood ejection. Moreover, strain analysis indicated that *Nlrx1^−/−^* heart synchronous contractility was compromised following exposures. Our findings demonstrated significantly reduced contractility and increased mechanical dyssynchrony across the endocardium, myocardium, and epicardium. These alterations collectively resulted in a significant decrease in TTP% in *Nlrx1^−/−^* hearts exposed to HT + UfCB + O_3_ along both the longitudinal and circumferential axes compared to *Nlrx1^+/+^* mice.

Growing evidence suggests that mitochondrial dysfunction is a major contributor to cardiac contractile dysfunction [81,82]. Mitochondrial dysfunction can reduce cardiac contractile performance by disturbing calcium homeostasis and impairing ATP-dependent calcium reuptake, thereby disrupting excitation–contraction coupling [83,84,85]. Owing to their dual genetic regulation, dynamic fission–fusion processes, and pronounced redox sensitivity, mitochondria are highly susceptible to a broad range of environmental toxicants [86,87,88].

NLRX1 deficiency results in significant and persistent mitochondrial dysfunction, as evidenced by reduced OCR in basal, state 3, uncoupled (state 3u), and state 4 respiration, which does not fully return to baseline even at 24 h post-exposure. As noted above, mitochondria were shown to be particularly vulnerable to HT exposure, and our findings highlight that their function was further compromised by UfCB + O_3_. At 2 h post-HT + UfCB + O_3_ exposure, *Nlrx1^−/−^* exhibited a more evident adaptive mitochondrial response characterized by increased complexes I and II activity, reflecting a compensatory mechanism to energetic stress. At this time point, cells appear to enhance electron transport chain efficiency to maintain ATP synthesis; however, complexes III, IV, and V are unable to sustain this response, and their activities were indeed significantly reduced post single or combined HT + UfCB + O_3_ exposure. On the other hand, *Nlrx1^+/+^* mice exhibited unaltered complexes I and II activity, with significantly reduced activity of complexes III, IV, and V, a pattern consistent with the conclusion of the early compensatory phase and the onset of metabolic alterations following acute environmental exposure. However, in *Nlrx1^+/+^* mice, complex activity largely returned to baseline values at 24 h post-exposure, except under HT + UfCB + O_3_ co-exposure. In this condition, *Nlrx1^+/+^* mice showed a significant increase in the activity of all five complexes, reflecting a stress-related alteration. In contrast, in *Nlrx1^−/−^* mice, UfCB + O_3_ single exposure and HT + UfCB + O_3_ co-exposure maintained significantly reduced activity of complexes III, IV, and V compared with *Nlrx1^+/+^*.

As mitochondrial function depends on strict metabolic balance, both diminished and elevated activity of the electron transport chain (ETC) can be detrimental, depending on the physiological context. Reduced ETC activity compromises ATP production and promotes energy deficits and disease, whereas excessive activity can increase ROS generation, leading to oxidative stress and cellular damage. Unlike *Nlrx1^+/+^* mice, *Nlrx1^−/−^* mice exhibited a persistent and marked reduction in the activity of complexes III, IV, and V up to 24 h post-exposure, even following UfCB + O_3_ alone, with further decline under combined stress conditions. These findings underscore the important role of NLRX1 in regulating responses to metabolic stress, potentially supporting an overall impairment in lung–heart crosstalk.

This work showed conceptual and technical innovations. Conceptually, it is novel in examining co-exposure to a realistic combination of UfCB + O_3_ and HT while elucidating a previously uncharacterized role of NLRX1 in mediating the interaction between these environmental stressors. Unlike most studies that focus on a single organ system, this work simultaneously assesses both pulmonary inflammation and cardiac physiological functions and biochemical changes following inhalation exposure. However, there are some limitations. Only a single sex of mice was studied; only a single HT level and air pollution concentration were used. We made these changes carefully to closely mimic the air pollution exposures, given that it was an expansive study design involving KO and WT mice, two organs, two time points, and four exposure groups. Since the main goal of this study was to assess how NLRX1 deficiency influences cardiopulmonary responses to combined heat and UfCB + O_3_ exposure, we aimed to minimize confounding factors in this initial mechanistic investigation. Using one sex allowed us to set up the model and identify the phenotype, and future studies will address the role of sex as a biological variable. Moreover, UfCB was chosen instead of PM_2.5_, and these materials differ in composition. This was done to reduce variability arising from particle source and seasonal differences, enabling the combination with a reactive gas to create a reproducible surrogate mixture for air pollution. Additionally, this decision was also driven by practical constraints, as the whole-body aerosol exposure system needed a substantial particle mass to maintain consistent exposures. Notably, these observations reflect an acute exposure scenario and provide initial evidence to guide future evaluations under chronic exposure conditions. To avoid exposing animal skin and introducing a variable heat exposure scenario, we did not perform baseline heart function measurements, which limits conclusions about heart function changes within the animal.

## 5. Conclusions

Collectively, these findings demonstrate that HT + UfCB + O_3_ co-exposure increases the risk of cardiac dysfunction. Our results indicate that acute exposure to HT and UfCB + O_3_ can impact heart function. They also suggest that NLRX1 plays a role in the heart’s adaptive response, since lacking NLRX1 changes the immediate physiological reaction to these environmental stresses. Further studies elucidating the underlying mechanisms of lung-to-heart crosstalk and the role of NLRX1 in heat stress response will be critically important given the projected rise in extreme heat events and additional risk factors for cardiac dysfunction, including AP, infections, chemical exposures, and an aging population.

## Data Availability

The original contributions presented in this study are included in the article/Supplementary Materials. Further inquiries can be directed to the corresponding author.

## Supplementary Materials

The following supporting information can be downloaded at: https://www.mdpi.com/article/10.3390/cells15181699/s1. This article has an online data supplement that contains (1) a data file containing raw data for all the figures and (2) values for all the ultrasound parameters, which are accessible from this issue’s table of contents online at the journal website.

## Abbreviations

The following abbreviations are used in this manuscript:

NLRX1	NOD-like receptor X1
HT	High temperature
UfCB	Ultrafine carbon black
O_3_	Ozone
PM_2.5_	Particulate matter with a diameter ≤ 2.5 µm
IFN	Interferon
dsRNA	Double-stranded RNA
NF-kB	Nuclear factor kappa B
MAVS	Mitochondrial antiviral signaling protein
NLRP3	NLR family pyrin domain containing 3
ERS	Endoplasmic reticulum stress
ELPI+	Electrical low-pressure impactor
APS	Aerosol particle sizer
SMPS	Scanning mobility particle sizer
MOUDI	Micro-Orifice Uniform Deposit Impactor
XPS	X-Ray Photoelectron Spectroscopy
CPC	Condensation particle counter
NO_2_	Nitrogen Dioxide
BAL	Bronchoalveolar lavage
TPA	Total protein assay
BCA	Bicinchoninic acid assay
PVDF	Polyvinylidene difluoride
HR	Heart rate
LV	Left ventricle
M-mode	Motion-mode
SV	Stroke volume
CO	Cardiac output
EF%	Ejection fraction percentage
FS%	Fractional shortening percentage
B-mode	Brightness-mode
TTP%	Time-to-peak percentage of strain
ETC	Electron transport chain
NADH	Nicotinamide Adenine Dinucleotide
ATP	Adenosine Triphosphate
XFe96	Agilent Seahorse XFe96 Extracellular Flux Analyzer
MAS	Mitochondrial assay solution
KH_2_PO_4_	Potassium dihydrogen phosphate
MgCl_2_	Magnesium Chloride
HEPES	4-(2-hydroxyethyl)-1-piperazineethanesulfonic acid
EGTA	Ethylene glycol-bis(β-aminoethyl ether)-N,N,N′,N′-tetraacetic acid
BSA	Bovine serum albumin
FCCP	Carbonyl cyanide 4-(trifluoromethoxy)phenylhydrazone
XF	Extracellular Flux
TMPD	N,N,N′,N′-tetramethyl-p-phenylenediamine
ADP	Adenosine diphosphate
OCR	Oxygen consumption rate
RT	Room temperature
ENDO	Endocardium
MYO	Myocardium
EPI	Epicardium
NAAQS	National Ambient Air Quality Standard
WHO	World Health Organization
OSHA	Occupational Safety and Health Administration
ROS	Reactive oxygen species
